# Postoperative morphologic changes of the isthmocele and clinical impact in patients treated by channel‐like (360°) hysteroscopic technique

**DOI:** 10.1002/ijgo.14387

**Published:** 2022-08-23

**Authors:** Paolo Casadio, Antonio Raffone, Andrea Alletto, Francesco Filipponi, Diego Raimondo, Alessandro Arena, Mariangela La Rosa, Agnese Virgilio, Camilla Franceschini, Giampietro Gubbini, Mario Franchini, Roberto Paradisi, Jacopo Lenzi, Antonio Travaglino, Antonio Mollo, Josè Carugno, Renato Seracchioli

**Affiliations:** ^1^ Division of Gynaecology and Human Reproduction Physiopathology IRCCS Azienda Ospedaliero‐Universitaria di Bologna Bologna Italy; ^2^ Department of Medical and Surgical Sciences University of Bologna Bologna Italy; ^3^ Department of Gynecology Madre Fortunata Toniolo Clinic Bologna Italy; ^4^ Department of Obstetrics and Gynecology Tuscany Health Agency Florence Italy; ^5^ Section of Hygiene, Public Health and Medical Statistics, Department of Biomedical and Neuromotor Sciences, Alma Mater Studiorum University of Bologna Bologna Italy; ^6^ Anatomic Pathology Unit, Department of Advanced Biomedical Sciences, School of Medicine University of Naples Federico II Naples Italy; ^7^ Gynecology and Obstetrics Unit, Department of Medicine, Surgery and Dentistry "Schola Medica Salernitana" University of Salerno Baronissi Italy; ^8^ Obstetrics, Gynecology and Reproductive Science Department University of Miami, Miller School of Medicine Miami Florida USA

**Keywords:** bleeding, cesarean section, cesarean section scar, defect, defect, hysteroscopy, isthmocele, metroplasty, niche, pain, treatment, uterus

## Abstract

**Objective:**

To evaluate the changes in (1) residual myometrial thickness (RMT), (2) cesarean scar defect (CSD) size, and (3) clinical symptoms, before and after channel‐like (360°) hysteroscopic resection for the treatment of CSD.

**Methods:**

A single‐center, observational, prospective, cohort study was carried out enrolling all symptomatic patients of childbearing age, diagnosed with CSD and routinely scheduled for channel‐like (360°) hysteroscopic resection from July 2020 to July 2021 at the Division of Gynecology and Human Reproduction Physiopathology, Department of Medical and Surgical Sciences, IRCCS Azienda Ospedaliero‐Univeristaria di Bologna. University of Bologna, Italy. The primary outcome was the difference in mean RMT before and after the procedure. Secondary outcomes were the differences before and 4 months after the surgery in: (1) CSD size measured by transvaginal ultrasound, (2) visual analog scale (VAS) scores for the symptoms, and (3) abnormal uterine bleeding (AUB) rate. Lastly, patients' satisfaction was assessed by the global impression of improvement (PGI‐I) score.

**Results:**

We found a significant difference before and after the procedure in: (1) mean RMT (+2.0 mm; *P* < 0.001); mean size of the CSD (base: +1.6 mm; height: −2.5 mm; transverse diameter: −3.2 mm; volume: −263.7 mm^3^; *P* < 0.001); (2) mean VAS score for dyspareunia (−5.84; *P* < 0.001), dysmenorrhea (−8.94; *P* < 0.001), pelvic pain (−2.94; *P* < 0.001); (3) AUB rate (91% vs. 3%; *P* < 0.001). Lastly, the mean PGI‐I score ± SD was 1.7 ± 0.9.

**Conclusion:**

Channel‐like (360°) hysteroscopic resection for the treatment of patients with symptomatic CSD may lead to an increase in RMT, decrease in CSD, and improvement of symptoms after the procedure, with high patient satisfaction.

## Synopsis

Channel‐like (360°) hysteroscopic resection for symptomatic cesarean scar defect may increase residual myometrial thickness, decrease defect height, transverse diameter, and volume, and improve symptoms.

## 1 INTRODUCTION

Cesarean scar defect (CSD) or isthmocele represents a frequent late complication of the most common surgical procedure performed on patients worldwide: the cesarean section (CS).^1^ Also known as niche, pocket, or diverticulum, it is defined as a reservoir‐like pouch defect on the anterior wall of the uterine isthmus or cervical canal located over the site of a previous cesarean section scar^2^ caused by alterations in the healing process of the hysterotomy site.^3^, ^4^, ^5^ The exact prevalence of isthmocele is unknown, however its clinical incidence is globally increasing along with the increase of the CS rate.^6^, ^7^ According to a recent review, the prevalence of isthmocele in randomly selected patients varies from 24% to 70% when evaluated with transvaginal ultrasound (TVUS).^1^

Patients with CSD are usually asymptomatic, but may complain of abnormal uterine bleeding (AUB) frequently described as postmenstrual spotting, dysmenorrhea, dyspareunia, and/or pelvic pain.^3^ The presence of CSD can lead to obstetric complications in subsequent pregnancies including placenta accreta, cesarean scar ectopic pregnancies and uterine rupture.^8^ CSD are diagnosed by TVUS and/or hysteroscopy or hysterosonography.^9^ However, the value of TVUS in patients with CSD aiming to identify patients at risk for uterine rupture remains unknown, and the causal link between obstetric complications and the presence of CSD requires further investigations.^10^, ^11^

Treatment of CSD ranges from expectant or medical management to surgical intervention including hysteroscopy, vaginal surgery, laparoscopy, as well as combined laparoscopic and hysteroscopic procedures. Despite the increased incidence of this challenging condition, there is no evidence in the literature of which is the best surgical technique for the treatment of CSD; furthermore, the characteristics of the CSD after surgical treatment have been poorly investigated. In particular, the TVUS assessment of residual myometrial thickness (RMT), defined as the shortest visible distance between the uterine serosa and the endometrium in the sagittal plane, is becoming increasingly important because it was hypothesized as a marker to assess the risk of uterine rupture during pregnancy and its thickness could have an important role when recommending the route and timing of delivery.^12^

Recently, we described the “channel‐like 360° technique” for the CSD treatment consisting in hysteroscopic resection not only of the fibrotic tissue underneath the niche, but also of the inflamed tissue placed around the niche and on the opposite site.^13^, ^14^

In this study, we aimed to evaluate changes in (1) RMT, (2) CSD size measured by TVUS, and (3) clinical symptoms, before and after channel‐like (360°) hysteroscopic resection for the treatment of patients with symptomatic CSD. Lastly, patients' satisfaction with the procedure was evaluated.

## 2 MATERIALS AND METHODS

### 2.1 Study protocol

The study was designed as a single‐center, observational, prospective, cohort study according to an “a priori*”* defined study protocol. The Strengthening the Reporting of Observational Studies in Epidemiology (STROBE) guidelines and checklist^15^ were followed for reporting the study.

We enrolled all consecutive symptomatic patients of childbearing age, diagnosed with CSD and scheduled for hysteroscopic surgery from July 2020 to July 2021 Division of Gynecology and Human Reproduction Physiopathology, Department of Medical and Surgical Sciences (DIMEC), IRCCS Azienda Ospedaliero‐Univeristaria di Bologna. University of Bologna, Italy. In fact, in our center, the channel‐like (360°) endocervical ablation is routinely offered by an experienced and dedicated surgeon as first line treatment for CSD.^13^, ^14^

All patients received extensive counseling on the risks, benefits and alternatives of the proposed treatment and voluntarily agreed to participate.

During the preoperative and postoperative evaluation, medical history and TVUS data were collected using an ad‐hoc case report form and reported in an electronic database. The presence of pelvic pain, dyspareunia and dysmenorrhea was assessed for each woman using a visual analogue scale (VAS), before and 4 months after the procedure. Patients were also asked about resolution of AUB, and satisfaction with the surgical procedure using the patient global impression of improvement (PGI‐I) score 4 months after treatment.^16^

### 2.2 Study outcomes

The primary outcome was the difference in mean RMT before and 4 months after surgery. Secondary outcomes were:

- the difference in CSD size before and after surgery, in term of mean size of CSD base, height, transverse diameter, and volume;
- the difference in VAS scores for pelvic pain, dyspareunia and dysmenorrhea before and after surgery.
- the difference in AUB rate before and after surgery; AUB consisted of intermenstrual bleeding (i.e. bleeding between cyclically regular onset of menses);
- the mean PGI‐I score; PGI‐I score assesses patient satisfaction on a scale from 1 (significantly much better) to 7 (significantly much worse), 4 months after the procedure.

### 2.3 Surgery

All patients were hysteroscopically treated with the “channel‐like (360°) technique” as previously described,^13^ by the same expert operator (P.C.). The procedure was performed in the operating room with the patients under general anesthesia, using the vaginoscopic approach with a 16 Fr mini resectoscope (Gubbini system, Tontarra, Medizintechnik, GmbH, Germany) with a 2.9 mm 0° lens and consists of four steps, as was recently reported by our group.^13^ Briefly, the first step is the resection of the proximal margin of the CSD using a 90° angled equatorial loop with bipolar energy setting on cutting mode at 100 W to eliminate the fibrotic tissue of the proximal edge of the niche. The second step is the resection of the distal margin to completely remove the fibrotic tissue of the distal margin at 360° (i.e., including all walls of the cervical canal), thus completing the endocervical ablation. The third step is performed using the “roller ball” electrode with energy settings on cutting mode at 100 W to obtain focal coagulation of the residual inflamed tissue present on the CSD surface and along the walls of the cervical canal. The fourth step consists in the control of the bleeding obtained with the ball electrode for coagulation of the small bleeding vessels at the level of the cervical canal. The reduction of the infusion pressure of the distension fluid facilitates the identification of these small vessels. All patients underwent surgery during the proliferative phase of their menstrual cycle.

### 2.4 Ultrasound

The diagnosis of CSD at the site of the previous CS(s) was carried out by TVUS. The preoperative and postoperative TVUS was always performed by the same experienced sonographer (A.A.) using Voluson E6 equipment (GE Healthcare, Chicago IL) with the 7.5 MHz vaginal probe. The CSD was identified in a two‐dimensional plane as an anechoic triangular area at the level of the cervical‐isthmic region of the anterior uterine wall. Ultrasound was performed according to a previously described technique.^17^, ^18^ Briefly, in the sagittal scan, the uterine version was documented (anteverted or retroverted), RMT measured, and the length and height of the niche were obtained. The depth of the niche was measured in the transversal plane. For each woman, the volume of the CSD was also calculated using the prolate ellipse formula: $\left(\text{length}∙\text{height}∙\text{depth}\right)x\;0.5233$, and the result was expressed in mm^3^.^17^, ^18^

The patients were also examined by TVUS 4 months after the surgery, in order to evaluate changes in the RMT. All TVUS were performed during the early proliferative phase of the menstrual cycle. Changes in the RMT width, mean size and volume of the CSD before and 4 months after the procedure were compared.

### 2.5 Sample size analysis

We used the estimates of Tsuji et al.^19^ to obtain our study parameters. We hypothesized the mean (±SD) RMT before surgery was 2.5 ± 1.5 mm, and the mean (±SD) RMT after surgery was 3.5 ± 2.0 mm. Assuming a correlation between the paired measurements of 0.7, the minimum sample size required to detect the resulting delta of 1 mm with a power of 80% and a significance level of 5% was 19. Although no clear evidence exists regarding the precise cut‐off of the residual myometrium thickness over CSD in predicting uterine perforation during hysteroscopic hystmoplasty or obstetrical complications (in particular uterine rupture during pregnancy), several Authors define it as crucial for these purposes.^20^, ^21^ Therefore, we believed that the gain of at least 1 mm could have clinical significance, and we adopted it for the sample size calculation.

### 2.6 Statistical analysis

Numerical variables were expressed as mean ± SD (range); categorical variables were expressed as numbers and percentages. Differences in RMT, CSD sizes and VAS scores of symptoms before and after surgery were studied with the paired *t*‐test for normally distributed variables and with the Wilcoxon signed‐rank test for non‐normally distributed variables. Normality was checked with Q–Q plot analysis and Shapiro–Francia test. Difference in AUB rate before and after surgery was studied with the McNemar's exact test. The distribution of symptoms according to the CSD volume were assessed with the Mann–Whitney U test. The significance level was set at 5% for all analyses. All data were analyzed using the Stata 15 software (StataCorp. 2017. *Stata Statistical Software: Release 15*. College Station, TX: StataCorp LP).

### 2.7 Ethical statement

The study received approval by the Institutional Review Board of the S IRCCS Azienda Ospedaliero‐Univeristaria di Bologna, S. Orsola Hospital (205/Oss/AOUBo) and it was carried out in accordance with the Declaration of Helsinki. All enrolled patients signed an informed written consent, and all data were deidentified to prevent the identification of the subjects.

## 3 RESULTS

### 3.1 Study population

During the study period, 32 patients were enrolled (Table 1). No patients dropped out of the study or lost to follow‐up. The number of previous CS ranged from 1 to 3, and AUB was the most common symptom. Other reported symptoms were pelvic pain, dyspareunia, and dysmenorrhea with a mean VAS of 3.0 ± 4.1, 5.8 ± 4.4 and 4.4 ± 4.5, respectively. Pelvic pain was directly correlated to the CSD volume (Table 2).

**TABLE 1:** Baseline characteristics of the study sample (*n* = 32).^a^

Characteristic	
Age, year	38 ± 4 (29–45)
Previous cesarean section	
1	15 (46.9)
2	14 (43.8)
3	3 (9.4)
Retroverted uterus	
No	12 (37.5)
Yes	20 (62.5)
Abnormal uterine bleeding	
No	3 (9.4)
Yes	29 (90.6)
Pelvic pain	
No	20 (62.5)
Yes	12 (37.5)
Dyspareunia	
No	12 (37.5)
Yes	20 (62.5)
Dysmenorrhea	
No	16 (50.0)
Yes	16 (50.0)

^a^ Data are presented as mean ± standard deviation (range), or as number (percentage).

**TABLE 2:** Cesarean scar defect volume (mm^3^) according to presence or absence of symptoms before surgery (*n* = 32).^a^

Symptom	CSD volume (mm^3^) by presence of symptom		*P* value
	Yes	No	
Abnormal uterine bleeding	503.9 ± 183.6	364.4 ± 83.1	0.232
	(202.0–954.0)	(309.7–460.0)	
Pelvic pain	661.1 ± 124.4	388.7 ± 122.3	<0.001
	(472.5–954.0)	(202.0–740.3)	
Dyspareunia	515.5 ± 188.4	449.8 ± 166.3	0.350
	(277.8–954.0)	(202.0–726.1)	
Dysmenorrhea	535.5 ± 200.6	446.2 ± 151.3	0.258
	(253.5–954.0)	(202.0–479.1)	

^a^ Data are presented as mean ± standard deviation (range).

### 3.2 Study outcomes

**FIGURE 1:**
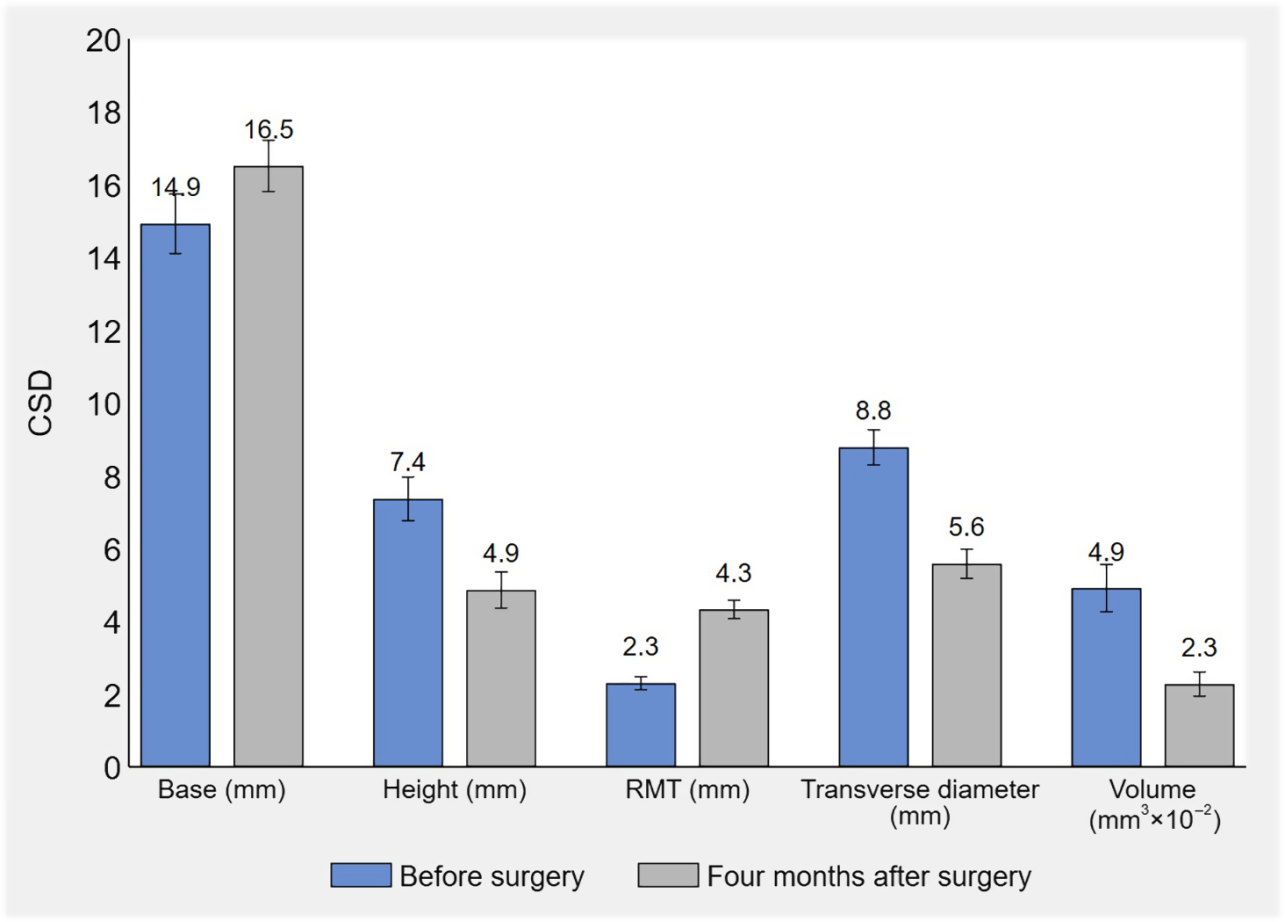
Mean estimates and 95% confidence intervals of residual myometrial thickness (RMT), and base, height, transverse diameter, and volume of the cesarean scar defect (CSD) before surgery and at 4‐month follow‐up (*n* = 32).

The mean preoperative RMT ± SD was 2.3 ± 0.3 mm, while the postoperative one was 4.3 ± 0.7 mm. As shown in Figure 1, there was a significant increase in mean RMT after surgery (+2.0 mm; 95% CI 1.9, 2.1; *p* value <0.001).

**FIGURE 2:**
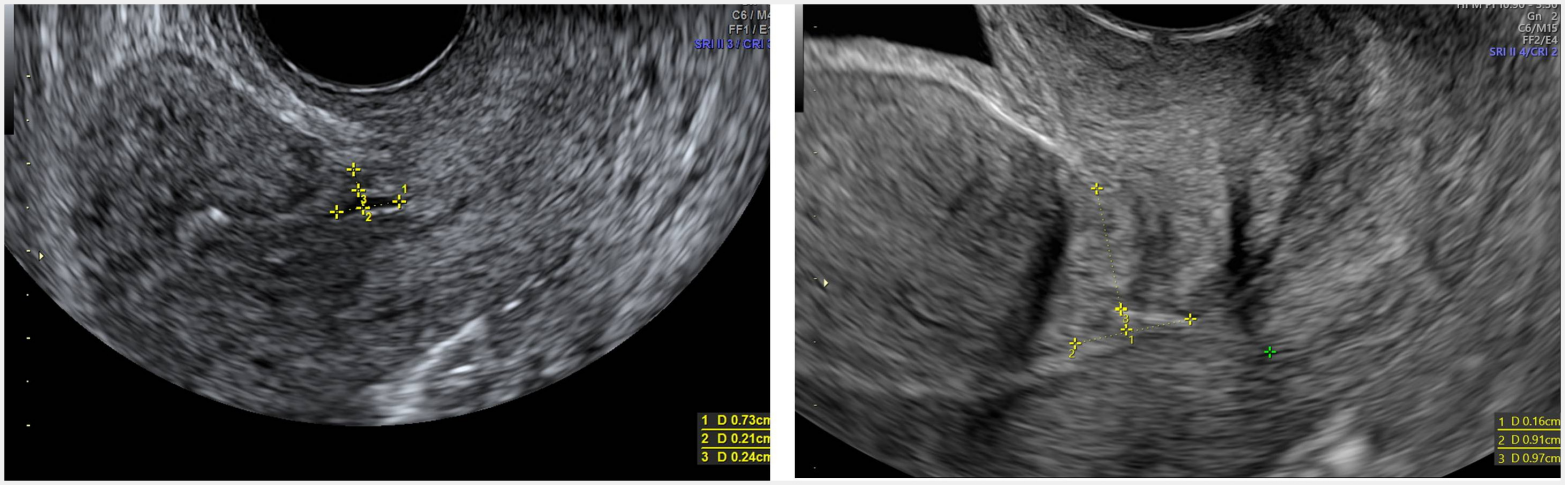
Preoperative and postoperative transvaginal ultrasound scan of cesarean scar defect (CSD). In the sagittal scan, calipers showed measurements of residual myometrial thickness (RMT), base and height diameter before surgery (left image) and at 4‐month follow up (right image).

There was a significant (P value <0.001) difference in all mean ± SD size measures of the CSD (base: +1.6 mm, 95% CI 1.4, 1.8; height: −2.5 mm, 95% CI –2.7, −2.3; transverse diameter: −3.2 mm, 95% CI –3.4, −3.0; volume: −263.7 mm^3^, 95% CI –299.1, −228.3; Figure 2).

No intra or post‐operative complications were observed, and patients were discharged the same day of the surgery.

Regarding symptoms, dyspareunia was complained in 20 (32%) patients before surgery, while all the patients denied any dyspareunia at 4 months after surgery. The mean VAS ± SD of dyspareunia decreased from 5.8 ± 4.4 to 0, with a mean difference of −5.84 (95% CI ‐7.41, −4.27; *P* value <0.001).

Dysmenorrhea was reported in half of the patients (50%) before surgery and in only 2 patients (6%) after surgery, with a mean VAS score ± SD of 4.4 ± 4.5 before and a mean VAS score ± SD of 0.1 ± 0.4 after, and a mean difference of −4.3 (95% CI ‐5.9, −2.7; *P* value <0.001).

The presence of pelvic pain was found in 12 patients (37%) before surgery, with a mean VAS score ± SD of 3.0 ± 4.1, and in only one (3%) woman after surgery, with a mean VAS score ± SD of 0.06 ± 0.3 and a mean difference of −2.9 (95% CI ‐1.5, −4.4; *P* value = 0.001).

Before surgery quite all the patients (90.6%) presented AUB and after surgery only one woman had persistent AUB (91% vs 3%; *P* value <0.001, Table 3).

**TABLE 3:** Variation in symptoms before and 4 months after surgery (*n* = 32).

Symptom	Before	After	Mean difference	*P* value
	surgery	surgery	(95% CI)	
Abnormal uterine bleeding	29 (90.6)	1 (3.1)	—	<0.001
Pelvic pain, VAS	3.0 ± 4.1	0.1 ± 0.4	−2.9 (−4.4 to −1.5)	0.001
Dyspareunia, VAS	5.8 ± 4.4	0.0 ± 0.0	−5.8 (−7.4 to −4.3)	<0.001
Dysmenorrhea, VAS	4.4 ± 4.5	0.1 ± 0.4	−4.3 (−5.9 to −2.7)	<0.001

Abbreviations: CI, confidence interval; VAS, visual analog scale.

All patients were satisfied with the surgical procedure, the mean PGI‐I score ± SD assessed 4 months after the procedure was 1.7 ± 0.9.

## 4 DISCUSSION

This study shows that, in patients who underwent channel‐like (360°) hysteroscopic resection for the treatment of symptomatic CSD, there was an increase in RMT, a change in size of the CSD (with a statistically significant decrease of CSD height, transverse diameter and volume), and an improvement of symptoms at 4 months after surgery. Moreover, all patients were satisfied with the surgical procedure.

Despite there is no evidence in the literature of which is the best approach for the treatment of CSD, treatment ranges from expectant or medical management to surgical intervention including hysteroscopic, vaginal and laparoscopic surgery or combined procedures. The vaginal surgery technique consists in the vaginal excision of the scar with consequent single layer or double layer suture of the uterus.^22^, ^23^ Instead, during laparoscopic approach, the scar can be opened by CO2 laser, monopolar scalpel, cold scissors or ultrasound knife, the fibrotic tissue is removed from the edges of the niche until healthy myometrium and uterine wall is then sutured in single or double layer.^24^, ^25^ Among hysteroscopic procedures, several techniques have been proposed to correct the proximal and/or distal margin of CSD plus or not the dome, including resectoscopic or office procedures.^26^, ^27^, ^28^, ^29^, ^30^, ^31^, ^32^, ^33^, ^34^, ^35^, ^36^ Recently, we developed the channel‐like 360° technique consisting in an minimally invasive, safe procedure for the treatment of patients with symptomatic CSD.^13^, ^14^ It restores the continuity of the niche with the cervical canal through the resection of the fibrotic and inflamed tissue at the level of all the uterine walls of the cervical‐isthmic region allowing normal menstrual flow through the cervix, as suggested by Thurmond et al.^37^ The improved clearance from the niche of inflamed material, menstrual flow and blood might explain the positive effect on pelvic pain and dyspareunia, besides a possible placebo effect. Moreover, the removal of the inflamed and congested scar tissue favors the re‐epithelialization of the cervico‐isthmic region with a new epithelium consisting of a single layer of cuboidal cells.^2^ This re‐epithelialization could be responsible for the reduction of bleeding at the level of the niche, favoring the resolution or improvement of AUB frequently reported by patients with CSD. These hypothetical effects might underlie a greater improvement in symptoms and other outcomes associated to the channel‐like 360° technique than other CSD surgical treatments; however, more comparative studies are necessary to draw conclusions.

Regarding RMT, several studies^6^, ^38^, ^39^ have indicated that it represents a key parameter in determining the choice of surgical technique to be used in symptomatic patients. Several authors^6^, ^38^, ^39^ have arbitrarily defined the minimum size of RMT below which resectoscopic isthmoplasty should not be recommended. In particular, Chang et al.^38^ suggested that the RMT must be greater than 2 mm in order to safely perform resectoscopic correction of the CSD. Furthermore, Li et al.^39^ increased this minimum recommended limit to 2.5 mm, and even to 3.5 mm in cases of patients desiring future fertility. Donnez et al.^6^ instead claimed that vaginal or laparoscopic repair would be preferable for patients with a RMT <3 mm who desire to conceive. However, in the literature, there is no reliable evidence correlating the minimum size of RMT with the risk of uterine perforation during the hysteroscopic procedure, neither with the obstetrical risk of uterine rupture during labor.^40^

To the best of our knowledge, our study is the first to evaluate postoperative changes in RMT using TVUS. Performing the channel‐like (360°) technique by an experienced surgeon, and treating anterior, posterior and lateral walls of the cervical canal, as opposed to only the uterine walls where the niche is located, causes an increase of the myometrial thickness also of the lateral walls generating a better result on the transverse diameter. In addition, we also observed changes in CSD size after surgery. We observed an increase in size of the base of the defect probably secondary to the removal of the fibrotic and congested tissue both at the level of the caudal and cranial margin of the CSD. Despite this, a greater reduction of CSD height and transverse diameter led to an overall decrease in the mean volume of CSD documented at 4 months after surgery. The CSD postsurgical volume reduction observed on TVUS in our study was greater than the one reported by Tsuji S et al.^19^ Noteworthy, the latter used magnetic resonance imaging as an evaluation method which might underlie discrepancies with our findings. In future studies, it could be interesting to assess a cohort of surgically treated CSD women by both ultrasound and magnetic resonance imaging. However, postsurgical changes in RMT and CSD size might have several explanations, such as the clearance of blood or inflamed material enlarging the niche, the elimination of the traction force exerted by the fibrotic tissue, the recovery of the full contractile activity of the muscular fibers lining the CSD. Our findings would support that RMT is not a static parameter, as for myometrial free margin during hysteroscopic myomectomy.^41^

Despite the prospective design and data collection with sample size analysis based on the primary outcome, our study has several limitations: (I) the lack of a sub‐stratification of AUB symptoms based on causes other than isthmocele; (II) the absence of a long‐term follow‐up evaluation which appears necessary to confirm our preliminary data on pain symptoms and AUB after hysteroscopic channel‐like resection; (III) the risk of ascertainment bias due to non‐blinded nature of the study; (IV) the single center experience which may limit generalizability of the results; (V) the lack of assessment of CSD morphology and changes though sonohysterography, which seems to provide better results in diagnosing CSD morphology compared to TVUS^42^; (VI) the absence of assessment of reproductive and obstetric outcomes, which, requiring a longer follow‐up, might be better evaluable through a retrospective study design; (VII) the lack of a multivariable regression analysis which could be useful to identify the patients more or less responsive to the treatment due to the small sample size; (VIII) the absence of data regarding the symptoms before CS(s), which made us unable to understand if they already existed or were caused by the presence of the subsequent isthmocele; (IX) a possible placebo effect on postoperative symptoms as patients were aware that the procedure was a treatment strategy; (X) a small sample size requiring confirmation of our findings on future larger series.

Although the current data are encouraging, the clinical relevance of the anatomical modifications of CSD and RMT has yet to be established. As shown by several studies,^17^, ^19^ the hysteroscopic technique of ishtmoplasty seems to have excellent implications in terms of restoring fertility, but longer follow up times are required to confirm its impact on the reproductive outcomes. Furthermore, the impact of each of the different surgical approaches and potential clinical implications in case of future pregnancy also needs to be further explored.

In conclusion, channel‐like (360°) hysteroscopic resection for the treatment of patients with symptomatic CSD may lead to an increase in RMT, a change in size of the CSD (with a decrease of CSD height, transverse diameter and volume), and improvement in clinical symptoms when evaluated 4 months after surgery with a high rate of patient's satisfaction with the procedure.

Further studies are needed to confirm these findings and to investigate their impact on fertility and pregnancy outcomes.

## AUTHOR CONTRIBUTIONS

PC contributed to study conception, study design, study methods, data extraction, data analysis, manuscript preparation, methods supervision; and whole study supervision. AR contributed to study conception, study design, study methods, data analysis, manuscript preparation, methods supervision; and whole study supervision. AA contributed to study conception, study design, study methods, data extraction, data analysis, and manuscript preparation. FF contributed to study design, study methods, data extraction, data analysis, and manuscript preparation. DR contributed to study conception, study design, study methods, data analysis, and manuscript preparation. AlA contributed to data extraction, data analysis, and manuscript preparation. MLR: study conception, data analysis, and manuscript preparation. AV contributed to study design, data extraction, and manuscript preparation. CF contributed to study design, data extraction, and manuscript preparation. GG contributed to study conception, study design, methods supervision, and manuscript preparation. MF contributed to methods supervision, study supervision, and manuscript preparation. RP contributed to study conception, study design, methods supervision, manuscript preparation, and whole study supervision. JL contributed to study conception, study design, data analysis, methods supervision, and manuscript preparation. AT contributed to study design, methods supervision, manuscript preparation, and whole study supervision. AM contributed to study design, methods supervision, manuscript preparation, whole study supervision. JC contributed to study conception, study design, methods supervision, manuscript preparation, whole study supervision. RS contributed to study conception, study design, methods supervision, manuscript preparation, and whole study supervision. All authors approved the final of the version to be published and agreed to be accountable for all aspects of the work in ensuring that questions related to the accuracy or integrity of any part of the work are appropriately investigated and resolved.

## CONFLICT OF INTEREST

The authors report no conflict of interest.

## Data Availability

Data sharing is not applicable to this article as no new data were created or analyzed in this study.
